# *α*-1-Acid Glycoprotein Concentration as an Outcome Predictor in Adult Patients with Sepsis

**DOI:** 10.1155/2019/3174896

**Published:** 2019-06-12

**Authors:** Sheng-Yuan Hsiao, Yun-Ru Lai, Chia-Te Kung, Nai-Wen Tsai, Chih-Min Su, Chih-Cheng Huang, Hung-Chen Wang, Ben-Chung Cheng, Yu-Jih Su, Wei-Che Lin, Yi-Fang Chiang, Jih-Yang Ko, Cheng-Hsien Lu

**Affiliations:** ^1^Department of Biological Science, National Sun Yat-Sen University, Kaohsiung, Taiwan; ^2^Department of Emergency Medicine, Chang Gung Memorial Hospital-Kaohsiung Medical Center, Chang Gung University College of Medicine, Kaohsiung, Taiwan; ^3^Department of Neurology, Chang Gung Memorial Hospital-Kaohsiung Medical Center, Chang Gung University College of Medicine, Kaohsiung, Taiwan; ^4^Department of Neurosurgery, Chang Gung Memorial Hospital-Kaohsiung Medical Center, Chang Gung University College of Medicine, Kaohsiung, Taiwan; ^5^Department of Medicine, Chang Gung Memorial Hospital-Kaohsiung Medical Center, Chang Gung University College of Medicine, Kaohsiung, Taiwan; ^6^Department of Radiology, Chang Gung Memorial Hospital-Kaohsiung Medical Center, Chang Gung University College of Medicine, Kaohsiung, Taiwan; ^7^Center for Shockwave Medicine and Tissue Engineering, Chang Gung Memorial Hospital-Kaohsiung Medical Center, Chang Gung University College of Medicine, Kaohsiung, Taiwan; ^8^Department of Neurology, Xiamen Chang Gung Memorial Hospital, Xiamen, Fujian, China

## Abstract

**Background:**

*α*-1-Acid glycoprotein (AGP) is an acute-phase protein that plays a role in first-line defense against infection and is therefore elevated in sepsis. We tested the hypothesis that AGP levels increase initially in sepsis and decrease after antimicrobial therapy and that these levels may predict treatment outcomes.

**Methods:**

AGP, biomarkers widely used in clinical practice, and maximum 24-h acute physiology and chronic health evaluation (APACHE)-II scores upon emergency department (ED) admission were prospectively evaluated and compared. We further examined changes in AGP concentrations 1, 4, and 7 days after admission and determined the value of AGP that may be used to accurately and reliably predict the prognosis in patients with sepsis.

**Results:**

Mechanical ventilation, white blood cell (WBC) counts, C-reactive protein (CRP) and lactate levels, maximum 24-h APACHE-II scores, and AGP concentrations were significantly higher upon admission in patients with sepsis who died. AGP and lactate concentrations were also significantly higher in non-survivors than in survivors on days 1, 4, and 7. As indicated by the stepwise logistic regression model analysis and area under the curve analysis, AGP was the best prognostic indicator, and the cut-off value for predicting fatality was 1307 *μ*g/mL, and any increase 1-ng/mL in AGP concentration would increase the fatality rate by 0.5%.

**Conclusion:**

Based on our observations, AGP may be a good prognostic predictor in patients with sepsis. In addition, serial AGP levels meet the requirements for predicting outcomes in patients with sepsis.

## 1. Introduction

Sepsis is a complicated syndrome resulting from the inappropriate expression of host factors in response to infection and is a major cause of death in patients that are hospitalized, in emergency departments (ED) and in critical care units [1]. In order to monitor the progression of sepsis in patients initially presenting at an ED and to accurately assess mortality after adequate treatment, reliable tools such as biomarkers or severity scores are needed for clinical use and to improve treatment outcomes. Previous studies have investigated certain commonly used detectable markers and disease severity scores to predict sepsis outcomes. However, these scores and biomarkers (e.g., white blood cell (WBC) and platelet counts, and C-reactive protein (CRP), lactate, and procalcitonin levels, among others) may be unreliable because of a lack of strict recruitment criteria or controversial results [2–7]. Therefore, more specific, more easily detectable biomarkers that are better than those currently used should be investigated.

*α*-1-Acid glycoprotein (AGP), also known as orosomucoid, is an acute phase protein that belongs to the immunoglobin family. It is an innate anti-inflammatory and immunoregulatory mediator that is involved in leukocyte extravasation, platelet aggregation, and endothelial permeability. Although the exact mechanisms have not been completely clarified, AGP displays anti-neutrophil and anti-complement activities in response to infection, inflammation, neoplasm, and tissue injury [8]. Few studies have explored the use of monitoring AGP in sepsis, and its role remains to be elucidated [7]. In particular, since the introduction of the new definition of sepsis in 2016 which was more stringent criteria of sepsis and better at predicting mortality than previous version [9], the prognostic manifestations of these biomarkers and the associated dynamic changes observed in patients with sepsis require further investigation. Through this prospective study, we tested the hypothesis that AGP may be used as a reliable predictor of prognosis and that its serial concentrations would increase initially after sepsis and decrease after disease control.

## 2. Patient Selection and Methods

### 2.1. Study Population and Definition

In this prospective study, we recruited 87 non-surgical and non-trauma adult patients with sepsis over a three-year period from January 2016 to August 2018 at Kaohsiung Chang Gung Memorial Hospital, an acute care teaching hospital. Our study was approved by the hospital's Institutional Review Committee on Human Research (no. 104-9397B and no. 103-5216B), and all participants (patients or legitimate relatives) provided written informed consent. For comparison, we also enrolled 39 sex- and age-matched healthy volunteers without clinical evidence of infection as the control group. Patients aged ≥ 18 years were screened daily and sepsis or septic shock was diagnosed according to the sepsis criteria defined by the Third International Consensus Definitions published in 2016 [10]. All patients with sepsis or septic shock enrolled in this study displayed sequential organ failure assessment (SOFA) scores that were ≥ 2 points higher than those associated with baseline status. Septic shock was defined as requiring vasopressors to maintain mean blood pressure above 65 mm Hg and serum lactate concentration above 18 mg/dL, with the presence of hypovolemia. Exclusion criteria included patients with (a) hematological disease and those receiving chemotherapy, (b) simultaneous comorbidities (such as combined tumors) that may have affected results, and (c) acute or chronic liver disease and (d) patients admitted 28 days priorly.

### 2.2. Clinical Assessment and Therapy

We collected demographic data and used standardized assessment scales to record clinical severity indexes as acute physiology and chronic health evaluation (APACHE)-II scores based on the worst physiological parameters recorded within the first 24 h of ED admission. Information about the infection source, antibiotic administration, and other treatments, including vasoactive agent supplementation, ventilator support, and renal replacement therapy, was recorded. Furthermore, consultation with an infectious disease expert to identify an appropriate antimicrobial treatment based on the guidelines for the infection cause during the first 24 h is an institutional practice.

### 2.3. Infectious Parameters and Clinical Severity Indexes

Sufficient blood was extracted via venipuncture of a forearm vein using an aseptic technique, and the blood specimen tubes were centrifuged at ambient temperature. The supernatant serum was then aliquoted and immediately shipped on dry ice to the Kaohsiung Chang Gung Memorial Hospital laboratory. The experiment was conducted after specimen preparation. All tests were conducted at the hospital's quality-controlled central laboratory. According to well-established methods, lactate levels and inflammatory markers such as WBC counts and differential counts, platelet counts, and CRP and procalcitonin levels were determined upon patient admission to the ED. APACHE-II scores were calculated according to the worst vital signs and laboratory data recorded within 24 h of ED admission. CRP levels were measured via enzyme immunoassay, procalcitonin levels were assessed via enzyme-linked fluorescent assay, and lactate levels were determined via serum-based assay. Other parameters, such as WBC counts and differential counts, and levels of creatinine (Cr), glutamic oxaloacetic transaminase (GOT), hemoglobin (Hb) were also conducted via internationally accepted laboratory methods.

### 2.4. Blood Sampling and Measurement of AGP

AGP concentrations were monitored after 24 h and blood was collected at follow-up time points on days 4 and 7. An interval of 72 h between time points was selected to increase the likelihood of changes in the levels of AGP and other mediators investigated being associated with acute phase protein alternation. AGP levels were measured using commercially available enzyme-linked immunosorbent assay (ELISA) kits (R&D Systems, Minneapolis, MN, USA).

### 2.5. Outcome Determination

Patients were divided into two groups (survival and non-survival groups) according to endpoint on 28-day mortality rate. Physicians evaluated the relationship between AGP concentration and mortality rate in patients with sepsis daily.

### 2.6. Statistical Analysis

Quantitative variables are reported as means ± standard deviation (SD) and continuous data were analyzed via Student's* t*-test. We used *χ*2 test or Fisher's exact test to analyze categorical variables expressed as rates (%). A correlation analysis was performed to explore the association between AGP concentration and WBC count, CRP, and lactate levels, as well as 24-h APACHE-II scores in patients with sepsis upon ED admission. An analysis of variance (ANOVA) was performed to compare AGP concentrations at three time points (days 1, 4, and 7). We used covariance analysis (ANCOVA) to compare groups and controlled for potential confounding variables. Moreover, we explored the association between significant variables and therapeutic outcomes and adjusted for potential confounding factors via stepwise logistic regression. Only variables strongly associated with sepsis prognosis (*p* <0.05) were included in the final model. Receiver operating characteristic (ROC) curves were drawn to measure the diagnosis and mortality performance of final significant parameters before determining cut-off values. The areas under the curve (AUCs) for each parameter were estimated and compared with respect to their diagnostic and prognostic capabilities in patients with sepsis. Statistical data analysis was performed using the SAS software package version 9.1 (2002, SAS Statistical Institute, Cary, NC, USA).

## 3. Results

### 3.1. Clinical Characteristics of Study Patients

A total of 87 patients with sepsis and 39 controls were recruited to the present study. The patients' demographic data revealed no significant differences with regard to underlying diseases such as hypertension, diabetes, and chronic heart disease (Table 1). However, there were significantly higher WBC counts and CRP levels, as well as lower platelet and Hb levels in the sepsis group than in the control group. In addition, serum AGP concentrations were significantly higher in patients with sepsis than in control participants (1137.9 ± 397.6 vs 524.3 ± 170.0, respectively;* p *< 0.001).

### 3.2. Correlation between AGP and Infection Parameters or Disease Severity Scores

The correlation between AGP, other inflammatory biomarkers, and clinical severity indexes upon ED admission was investigated and the statistical test results (correlation coefficients and* p*-values) are listed in Table 2. Mean AGP concentrations were significantly associated with CRP levels (*γ* = 0.53,* p *< 0.01), although no significant correlation between AGP concentrations and WBC counts (*γ* = 0.04,* p *= 0.73), lactate levels (*γ* = 0.13,* p *= 0.27), or maximum 24-h APACHE-II scores (*γ* = 0.21,* p *= 0.06) was observed.

### 3.3. Comparisons of Clinical Manifestations between Sepsis and Septic Patients

The clinical characteristics in patients with sepsis (n = 16) and septic shock (n = 71), including potential diseases, clinical manifestations, disease severity scores, ventilator and inotropic agent use, and some laboratory data, are listed in Table 3. Among the 87 studied group, 81.6% (71/87) experienced septic shock within 24 h of admission. No significant difference in age, gender, chronic diseases, maximum 24-h APACHE II scores, mortality ratio, or most infection parameters was observed. Only CRP showed significant higher in septic shock group than sepsis group. There was also no marker difference in AGP concentrations between sepsis and septic shock patients.

### 3.4. Comparison of the Characteristics of Survivors and Non-Survivors with Sepsis

The clinical characteristics of patients in the survival and non-survival groups, including potential diseases, clinical manifestations, disease severity scores, mortality rates, and laboratory data, are listed in Table 4. Among the 68 survivors with sepsis, 77.9% (53/68) experienced septic shock within 24 h of admission, and among the 19 non-survivors, 94.7% (18/19) had progressed to septic shock. Moreover, 39.7% (27/68) of patients in the survival group and 31.6% (6/19) of patients in the non-survival group showed bacteremia. A significantly higher maximum 24-h APACHE-II score was observed in non-survivors than in survivors (23.5 ± 9.1 vs 19.1 ± 6.7,* p *= 0.02). Non-survivors showed significantly higher serum levels of WBC, CRP, lactate, and AGP upon admission than survivors (respectively 17.9 ± 8.6 vs 13.5 ± 7.5,* p *< 0.05; 216.8 ± 89.6 vs 159.6 ± 110.0,* p *= 0.02; 39.1 ± 13.2 vs 29.1 ± 10.3,* p *< 0.01; and 1491.8 ± 449.2 vs 1039.0 ± 321.8,* p *< 0.01). The indicators mentioned above suggest an increasing tendency in the non-survivors in contrast to survivors. In addition, the percentage of mechanical ventilation was higher in non-survivors than survivors (63.2% (12/19) vs 26.5% (18/68); odds ratio, OR (95% confidence interval, CI): 4.76 (1.62-13.9),* p *= 0.01). The use of steroid and vasoactive agents did not differ remarkably between non-survivors and survivors.

### 3.5. Time Course of Circulating AGP and Lactate Concentrations in Survivors and Non-Survivors

**Figure 1:**
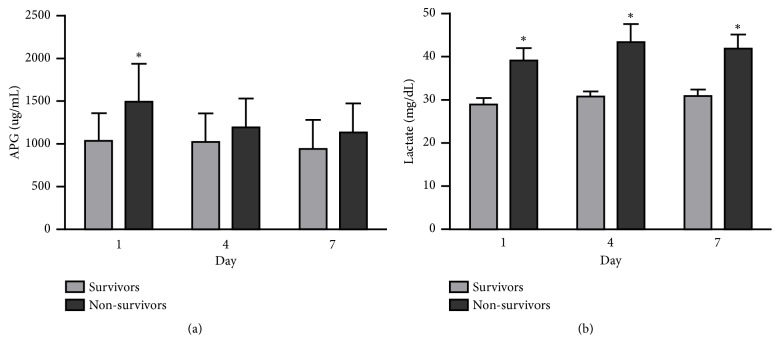
Concentrations of (a) *α*-1-acid glycoprotein (AGP) and (b) lactate on days 1, 4, and 7 between survivors and non-survivors. *∗p *< 0.05, survivors vs. non-survivors.

Circulating AGP and lactate concentrations were monitored in all patients with sepsis on days 1, 4, and 7. Dynamic changes in the serum AGP and lactate levels of both survivors and non-survivors are presented in Figure 1. Non-survivors showed significantly higher AGP concentrations than survivors on day 1 (1491.8 ± 449.2 vs 1039.0 ± 321.8,* p *< 0.01), but no significant differences were observed on day 4 (1190.1 ± 338.7 vs 1021.3 ± 331.7,* p *= 0.09) or day 7 (1036.1 ± 335.2 vs 943.7 ± 335.0,* p *= 0.07). Lactate concentrations were significantly higher in non-survivors than survivors on day 1 (39.1 ± 13.2 vs 29.1 ± 10.3,* p *<0.01), day 4 (43.3 ± 17.7 vs 31.0 ± 6.4,* p *= 0.01), and day 7 (41.9 ± 11.9 vs 31.1 ± 7.1,* p *<0.01). However, repeated measurements using ANOVA with Scheffe's method of multiple comparison revealed that AGP and lactate concentrations differed significantly between non-survivors and survivors at days 1, 4, and 7 (*p *< 0.01).

### 3.6. Predictive Factors of Clinical Outcomes

**Figure 2:**
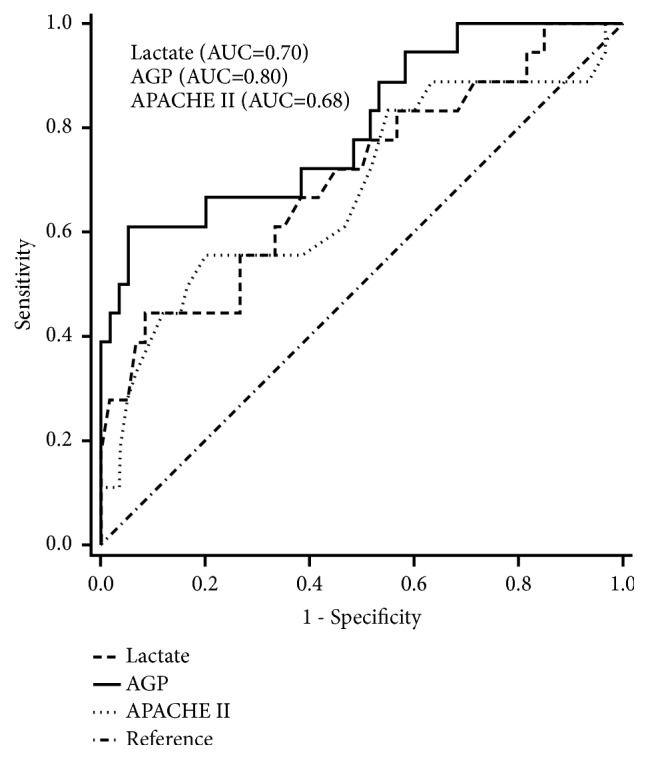
Receiver operator characteristic curve for serum AGP and lactate levels and maximum 24-h acute physiology and chronic health evaluation (APACHE) II scores.

Of the 87 patients with sepsis enrolled, 19 (21.8%) died in the hospital. The potential prognostic performances of the 87 patients with sepsis are listed in Table 4. Statistical analysis of the clinical manifestations and laboratory data between the survivors and non-survivors upon admission yielded the following results: maximum 24-h APACHE-II scores,* p* = 0.02; WBC count,* p* < 0.05; CRP concentration,* p* = 0.02; lactate concentration,* p* < 0.01; AGP concentration,* p* < 0.01; and mechanical ventilation,* p* = 0.01. Results from a stepwise logistic regression model including significant variables, age, and sex showed that serum AGP and lactate levels, as well as maximum 24-h APACHE-II scores upon admission, were independently correlated with sepsis outcome. The effectiveness of infection markers in detecting sepsis prognostic capability in the ED was assessed via AUC analysis. The AUC for AGP and lactate levels and maximum 24-h APACHE-II scores were 0.80 (95% CI: 0.68-0.93;* p* < 0.01), 0.70 (95% CI: 0.56-0.85;* p* = 0.01), and 0.68 (95% CI: 0.53-0.84;* p* = 0.02), respectively, indicating that AGP levels performed best as a predictor of sepsis prognosis, with lactate as second best. Moreover, the cut-off AGP and lactate concentrations for predicting sepsis fatality were 1307.0 *μ*g/mL (sensitivity = 66.7% and specificity = 80.0%) and 33.75 ng/mL (sensitivity = 66.7% and specificity = 61.7%), respectively (Figure 2). A 1-ng/mL increase in AGP and lactate concentrations would increase the fatality rate by 0.5 % and 10.5%, respectively.

## 4. Discussion

Through this study, we confirmed the hypothesis that AGP levels initially increase during sepsis and decrease after antimicrobial therapy and that AGP levels can predict treatment outcomes.

The present study assessed serial changes in the acute phase protein biomarker AGP in patients with sepsis and yielded six major findings. First, patients with sepsis exhibited significantly higher serum WBC counts, as well as AGP and CRP levels, and lower Hb and platelet levels than healthy volunteers. Second, AGP concentrations were positively correlated with CRP levels upon ED admission. Third, CRP showed significantly higher concentrations in septic shock patients than sepsis patients. Fourth, there was a significantly higher percentage of patients requiring mechanical ventilation, and higher maximum 24-h APACHE-II scores, WBC counts, and CRP, lactate, and AGP concentrations in the non-survivor group than the survivor group. Fifth, serial AGP and lactate levels increased significantly in non-survivors from day 1 to 7. Lastly, a stepwise logistic regression model analysis revealed that maximum 24-h APACHE-II scores and AGP and lactate levels were independent prognostic factors, and AUC analysis showed that AGP levels performed the best as predictors of fatality. The AGP concentration cut-off value for predicting fatality was 1307.0 *μ*g/mL (sensitivity = 66.7% and specificity = 80.0%), and a 1-ng/mL increase in AGP concentration would increase the fatality rate by 0.5%.

The main aim of this study was to investigate the use of monitoring changes in AGP levels upon ED admission and thereafter in predicting clinical outcomes, in comparison to conservatively used infection indicators. With regard to physiopathological mechanism, AGP is primarily synthesized in hepatocytes and its concentration in circulation increases 2- to 7-fold during the acute phase reaction, in response to systemic tissue injury, inflammation, and infection. During sepsis, AGP promotes the anti-inflammatory response by binding to the adhesion molecule L-selectin, decreasing neutrophil migration and rolling, granulocyte extravasation, and recruitment of T-cells to the endothelium or of platelets to the infection site, and modulating nitro oxide-dependent pathways and glycan moiety composition [8, 11, 12]. AGP gene expression is controlled by several mediators, such as glucocorticoids and cytokine modulators primarily involving interleukin (IL)-1L-1, IL-6, tumor necrosis factor-kinoid (TNF-K), and IL-6-associated cytokines [13, 14]. In addition, AGP may exert protective effects during infection by inhibiting lipopolysaccharide (LPS) toxicity and promoting LPS clearance from the body by binding with it to form AGP-LPS complexes [15]. In a study by McNamara et al., AGP was shown to weaken the proinflammatory effects of highly conserved bacterial lipoid A molecules and cytokines such as platelet-activating factor, thus demonstrating its protective properties [16].

In this study, a significantly higher percentage of mechanical ventilation was observed in non-survivors than survivors (OR [95% CI]: 4.76 [1.62-13.9],* p *= 0.01) and this may indicate an increase in sepsis severity. Mechanical ventilation may therefore be considered a risk factor or indicator of sepsis severity. It is used to prevent lung collapse and decrease sepsis-induced acute lung injury, acute respiratory distress syndrome, and acute respiratory failure in severe sepsis [17, 18].

In the present study, AGP and CRP levels were significantly correlated in patients with sepsis upon admission, and this observation was similar to that reported in previous studies [6, 19]. AGP and CRP are both hepatocyte-secreted positive acute phase proteins, with the former playing an important role in human infection. Both exert protective effects as plasma concentrations increase during early sepsis [20]. We inferred that the relationship between AGP and CRP resides in the fact that they are secreted by the same cytokines, such as IL-1, IL-6, and glucocorticoids, to promote anti-inflammatory effects during early sepsis. In a group of iron-deficient participants, AGP and CRP were associated during chronic infection [13]. However, there are no studies directly exploring the association between these two biomarkers in acute infection and further trials may therefore be needed.

Our study demonstrated that only serum AGP and lactate levels, as well as maximum 24-h APACHE-II scores, were independent indexes to predict sepsis outcomes as was shown by a stepwise logistic regression model after adjusting for age, gender, CRP levels, and WBC counts. We further analyzed AGP and lactate levels, and maximum 24-h APACHE-II scores via ROC curve analysis and found that AGP and lactate levels were both significant and were better predictors of sepsis outcome. However, AGP was the best prognostic predictor, and this observation is in agreement with that reported in previous studies [7, 21, 22]. However, in a 96-h early mortality survey, which is different to our 28-day outcome study, AGP levels were lower in non-survivors than survivors, which are inconsistent with our observations [6].

In a dynamic analysis of AGP levels in different outcomes, we observed that AGP concentrations differed significantly at different time points in survivors and non-survivors and that patients who died showed higher AGP concentrations. This is because AGP exerts protective effects during early sepsis, but may become damaging if high levels are maintained over an extended period of time, resulting in immunoparalysis during the later phase of sepsis [23]. Critical and uncontrolled sepsis would provoke uncontrollable inflammation and persistently high AGP concentrations, subsequently leading to damage and increased mortality. This may result from AGP binding-induced constitutional changes or inhibition and may impair the aggregation of neutrophils at infectious foci [24]. Therefore, dynamic AGP concentrations may be used as accurate estimates to monitor the outcome of inflammation or infection. This is consistent with the observation that AGP concentrations increase slowly and steadily (reaching peak level and persisting for 5-6 days) in response to inflammation and infection and thus reflect disease progression [25, 26]. Constantly high AGP concentrations in patients with sepsis suggest poor prognosis and indicate the need for more aggressive treatment.

In addition, we also observed that lactate levels showed good prognostic predictive capacity. During sepsis, increased lactate concentrations were attributed to anaerobic metabolism development, glycolysis, catecholamine-related Na-K pump activity stimulation, pyruvate dehydrogenase activity alternation, and lactate clearance. Several studies have revealed lactate levels as predictive indicators of tissue perfusion and mortality rate [27, 28]. Certain studies reported changes in lactate levels in early inflammation or infection, especially within 6 h of admission as lactate is reduced to 50% of its original concentrations in 1 h, directly reflecting disease severity and mortality rate [29, 30]. Changes in lactate levels indicate a difference in production and clearance during sepsis. Serial lactate measurements may be more effective indexes than single values in prognostic prediction, and non-survivors showed constantly higher lactate concentrations in sepsis evolution than survivors in our study. However, changes in lactate levels did not contribute to mortality prediction. AGP is a better prognostic predictor than lactate and it is a practical and convenient biomarker as it only requires blood to be drawn once upon ED admission.

Several studies have investigated the role of APACHE II score as a prognostic predictor in outcome of patients with sepsis [31–35]. One study enrolled those patients, who admitted to the intensive care unit (ICU) and met the diagnostic criteria for Sepsis-3, and these patients were divided into survival group and death group and found the AUC for APACHE II in those patients with worse outcome was 0.68 [31]. The other study enrolled those patients with severe sepsis or septic shock and tried to determine and compare the predictive ability of each marker for the risk of unfavorable evolution (in-hospital, 28-day, and 90-day mortality) and found the AUC for 28-day mortality for APACHE II score was 0.618 [32]. The other retrospectively study enrolled those septic patients who admitted to ICU and compared APACHE II and APACHE III scores in predicting hospital mortality [33]. The study demonstrated APACHE II was as good as APACHE III in predicting hospital mortality and the AUC of APACHE II in predicting hospital mortality was 0.8 [33]. The other study validates the role of APACHE II score at 24 hours after admission in predicting mortality in urosepsis [34]. The study enrolled those patients who had more severity of sepsis with higher APACHE II score (24.31 ± 6.48 in survivors and 32.39 ± 5.09 in those expired) than ours (19.1 ± 6.7 in survivors and 23.5 ± 9.1 in non-survivors) and found AUC of APACHE II score was 0.760. Another study enrolled those patients with sepsis/severe sepsis/septic shock and predicted 28-day mortality by using scoring systems for septic patients in an ICU setting [35]. The enrolled patients had been associated with higher proportion of comorbidities (dementia, hepatic cirrhosis, hematologic malignancy, and metastatic tumor) and were elder than ours, and AUC of APACHE II score (0.756) was also higher than ours. The discrepancy between these studies and our work may be attributed to differences in diagnostic criteria for sepsis (e.g., sepsis 2 and 3) and severity of sepsis, timing of APACHE II evaluation (e.g., At ER or admitted to ICU), outcome predictions (e.g., in-hospital, 28-day, and 90-day mortality ), and statistical methods. APACHE-II gives less validity in new sepsis criteria patients due to less important in above data in new version of sepsis trend to enrolled organ dysfunction patients [36]. In this present study, AGP increased and directly reflected the cellular and metabolic dysfunction and provided a better prognostic performance than 24-h APACHE II score.

Although our study demonstrated that AGP levels upon admission could be powerful predictors of mortality rate in patients with sepsis, certain limitations must be considered. First, AGP levels may be influenced by both age and gender, and levels may be higher in elderly patients. Our study included elderly participants, inevitably leading to higher sepsis incidence and fatality rates. Second, we monitored serial AGP and lactate level changes on days 1, 4, and 7 in this study. Biomarker measurement intervals to evaluate the relationship between therapeutic outcomes and changes in biomarkers should be individualized according to the half-life of each biomarker, which may differ. Further studies including shorter time intervals should be conducted in the future.

## 5. Conclusions

Our results indicate that AGP is a more valuable prognostic predictor during sepsis than other widely used indicators and severity scores, such as WBC counts, APACHE-II scores, and CRP, lactate, and procalcitonin levels. Serial AGP level measurements meet the major requirements for outcome prediction in treating patients with sepsis according to the new definition adopted in 2016. These findings indicate that AGP concentrations may be used to guide therapy and monitor disease severity and mortality in patients with clinical sepsis.

## Figures and Tables

**Table 1:** Clinical characteristics of patients with sepsis and control subjects.

	Control subjects	Sepsis patients	p value
	n = 39	n = 87	
Age (y) (mean ± SD)	56.4 ± 12.0	64.3 ± 13.9	NS
Male (%)	61.5	67.8	NS
Potential disease			
Diabetes (%)	0	37.9	<0.01*∗*
Hypertension (%)	10.2	50.1	<0.01*∗*
Chronic heart disease (%)	0	11.5	<0.01*∗*
Laboratory data (mean ± SD)			
White blood cells (×10^9^/L)	5.6 ± 2.0	14.4 ± 7.8	<0.001*∗*
Platelet (×10^4^/L)	222.6 ± 62.0	177.5 ± 104.5	<0.001*∗*
Hemoglobin(mg/dL)	14.1 ± 1.8	11.9 ± 2.2	<0.01*∗*
C-reactive protein (mg/L)	1.2 ± 1.0	170.0.1 ± 118.2	<0.001*∗*
AGP (*μ*g/ml)	574.3 ± 170.0	1140.8 ± 399.3	<0.001*∗*

SD, standard deviation; NS, not significant; AGP: *α*-1-acid glycoprotein; *∗*p<0.05

**Table 2:** Correlation analysis between *α*-1-acid glycoprotein (AGP) levels, other biomarkers, and clinical severity indexes.

Variables	*α*-1-Acid glycoprotein	
	r	P value
White blood count	0.04	0.73
C-reactive protein	0.53*∗*	<0.01
Lactate	0.13	0.27
APACHE score	0.21	0.06

SD, standard deviation; APACHE, Acute Physiology and Chronic Health Evaluation; *∗* = P<0.05

**Table 3:** Comparisons of clinical features between sepsis and septic shock patients.

	Sepsis	Septic shock	p value
	n = 16	n = 71	
Age (y) (mean ± SD)	63.8 ± 16.0	65.5 ± 14.8	0.69
Male/Female (n)	13/3	46/25	0.20
Potential diseases [n (%)]			
Diabetes	4 (25.0)	24 (33.8)	0.50
Hypertension	8 (52.0	37 (52.1)	0.88
Chronic lung disease	2 (12.5)	7 (9.9)	0.75
Cerebrovascular disease	3 (18.8)	9 (12.7)	0.52
Chronic heart disease	2 (12.5)	3 (4.2)	0.20
Chronic renal disease	3 (18.8)	19 (26.8)	0.51
Clinical presentations (mean ± SD)			
Systolic BP (mmHg)	140.1 ± 31.1	85.6 ± 25.7	P<0.01
Heart rate (bpm)	113.7 ± 28.7	109.4 ± 25.5	0.56
Clinical severity index (mean ± SD)			
Maximum 24-h APACHE II score	17.8 ± 5.3	20.6 ± 7.8	0.17
Bacteremia [n (%)]	5 (31.3)	28 (39.4)	0.54
Intervention within 24 hours [n (%)]			
Mechanical ventilator	5 (31.3)	25 (35.2)	0.76
Vasoactive agent	0 (0)	6 (8.5)	0.37
Expired [n (%)]	1 (6.3)	18	0.10
Laboratory data (mean ± SD)			
White blood cells (×10^9^/L)	12.0 ± 6.7	15.0 ± 8.0	0.18
Hemoglobin (mg/dL)	11.4 ± 1.5	12.0 ± 2.3	0.32
Platelet counts (×10^4^/L)	205.2 ± 122.8	171.3 ± 100.0	0.24
C-reactive protein (mg/L)	113.9 ± 86.7	182.0 ± 109.0	0.02*∗*
Lactate (mg/dL)	27.0 ± 10.8	32.6 ± 11.9	0.10
Cr (mg/dL)	2.1 ± 2.6	2.7 ± 2.5	0.41
Procacitonin (ng/mL)	9.1 ± 8.9.	28.0 ± 18.6	0.67
AGP (ng/ml)	1080.1 ± 354.0	1150.9 ± 408.3	0.52

SD, standard deviation; APACHE, Acute Physiology and Chronic Health Evaluation

**Table 4:** Association between AGP levels and other manifestations in patients with sepsis (survivors and non-survivors).

	Survivors	Non-survivors	Crude OR	p value	Adjusted OR	p value
	n=68	n=19	(95% CI)		(95% CI)	
Age (y) (mean ± SD)	64.0 ± 15.4	69.5 ± 12.3		0.16	−	
Male/Female (n)	45/23	14/5	1.43 (0.46-4.47)	0.59		
Potential diseases [n (%)]						
Diabetes	19 (27.9)	9 (47.4)	2.32 (0.82-6.60)	0.16		
Hypertension	36 (52.9)	9 (47.4)	0.80 (0.29-2.22)	0.80		
Chronic lung disease	7 (10.3)	2 (10.5)	1.02 (0.20-5.40)	1.00		
Cerebrovascular disease	9 (13.2)	3 (15.8)	1.23 (0.30-5.08)	1.00		
Chronic heart disease	3 (4.4)	2 (10.5)	2.55 (0.39-16.5)	0.64		
Chronic renal disease	15 (22.1)	7 (36.8)	2.06 (0.69-6.16)	0.24		
Clinical presentations (mean ± SD)						
Systolic BP (mmHg)	95.3 ± 33.8	96.8 ± 35.9		0.87		
Heart rate (bpm)	109.2 ± 25.5	114.0 ± 28.3		0.48		
Shock within 24 hours [n (%)]	53 (77.9)	18 (94.7)	5.09 (0.63-41.3)	0.18		
Clinical severity index (mean ± SD)						
Maximum 24-h APACHE II score	19.1 ± 6.7	23.5 ± 9.1		0.02*∗*	1.13 (1.01-1.26)	0.04
Bacteremia [n (%)]	27 (39.7)	6 (31.6)	1.15 (0.24-2.01)	0.70		
Intervention within 24 hours [n (%)]						
Mechanical ventilator	18 (26.5)	12 (63.2)	4.76 (1.62-13.9)	0.01*∗*		
Vasoactive agent	5 (71.4)	1 (14.3)	0.07 (0.01-0.97)	0.10		
Laboratory data (mean ± SD)						
White blood cells (×10^9^/L)	13.5 ± 7.5	17.9 ± 8.6		<0.05		
Hemoglobin (mg/dL)	11.9 ± 2.0	11.9 ± 2.8		0.98		
Platelet counts (×10^4^/L)	182.6 ± 109.6	159.5 ± 84.0		0.40		
C-reactive protein (mg/L)	156.9 ± 110.0	216.8 ± 89.6		0.02*∗*		
Lactate (mg/dL)	29.1 ± 10.3	39.1 ± 13.2		<0.01*∗*	1.105 (1.02-1.2)	0.01
Cr (mg/dL)	2.1 ± 1.6	2.6 ± 2.4		0.28		
GOT (mg/dL)	57.01 ± 41.8	60.6 ± 49.6		0.77		
Procacitonin (mg/dL)	24.0 ± 26.7.	28.4 ± 32.2		0.67		
AGP (ng/ml)	1039.0 ± 321.8	1491.8 ± 449.2		<0.01*∗*	1.005 (1.002-1.008)	<0.01

SD, standard deviation; APACHE, Acute Physiology and Chronic Health Evaluation

## Data Availability

The data used to support the findings of this study are available from the corresponding author upon request.
